# SPIE: An organizational framework to facilitate accessibility in academic statistical consulting centers

**DOI:** 10.1017/cts.2026.10712

**Published:** 2026-02-13

**Authors:** Marianne Huebner, Michael Wallace, Felesia Stukes, Sam Manski, Maria Montez-Rath

**Affiliations:** 1 Statistics and Probability, Michigan State University, USA; 2 Center for Statistical Training and Consulting, Michigan State University, USA; 3 Department of Statistics and Actuarial Science, University of Waterloo, Canada; 4 Department of Computer Science, Engineering, and Mathematics, Johnson C Smith University, USA; 5 Data Innovation Lab, Tech Impact, USA; 6 Quantitative Sciences Unit, Stanford University School of Medicine, USA; 7 Division of Nephrology, Stanford University, USA

**Keywords:** Statistical consulting, accessibility, neurodivergence, Universal Design for Learning, accommodation

## Abstract

Academic statistical consulting centers collaborate and provide statistical support to faculty and graduate students for their research projects. Clients seeking statistical support may face communication and cognitive accessibility barriers that consultants may not be aware of. Many researchers do not disclose their personal circumstances, such as neurodivergent behaviors. Universal Design for Learning is a framework that aims to support individuals regardless of their abilities or learning styles, by providing multiple means of engagement, presentation, and action and expression. Adapting these principles for statistical consulting and taking time to assess specific needs of individuals helps improve the experiences of clients and consultants and leads to high quality statistical support and successful completion of research projects. We propose an organizational framework applicable to statistical consulting environments aligned with accessibility guidelines to improve access and empower clients and consultants. Providing a respectful environment where differences are normalized and welcomed creates a sense of belonging for everyone.

## Introduction

Academic statistical consulting centers (SCC) collaborate with faculty and graduate students to provide expert statistical support, enhance research quality, and foster methodological rigor for successful project outcomes. This may include providing guidance on study design, statistical methods, research data management, statistical software, conducting statistical analyses, help with presentation and interpretation of results, and collaborating on manuscripts and grants. Throughout this paper we use the terms statistical consulting, clients, and consultants for ease of reading. Other terms, such as statistical or data science programs, domain experts, and statistical collaborators are also common. A consultant-client relationship can be similar to a collaborative relationship. While collaborators are typically involved throughout a research project, consultants may only complete specific tasks at specific times. As such, establishing long-term accessibility practices within a collaborative team may prove easier than within a consulting context where contact between individuals is more sporadic. However, accessibility principles apply universally, and even in the collaborative setting, periodic “check-ins” to ensure accessibility needs are met should be considered.

Consulting requires balancing the demands of concurrent projects necessitating strong organizational and time-management skills. Topics are rarely routine, and statistical consultants’ tasks include educating domain experts about recent developments in statistics or explaining potential pitfalls that may be an issue for a research study [1].

The aim of this paper is to discuss challenges regarding communication and cognitive accessibility for statistical consulting and collaborations and provide strategies for improvement. We propose an organizational, multi-faceted framework for SCCs to support accessibility, SPIE, which stands for Staff training, Processes and Practices, Infrastructure, and Evaluation. The paper is organized as follows. First, we present the organizational framework. We suggest specific strategies aligned with Universal Design for Learning (UDL) and discuss how these benefit a wide range of individuals, both clients and consultants. Then, we present aspects of this organizational framework regarding effective communication, meeting modalities, and web accessibility. Finally, we illustrate accessibility needs for neurodivergent individuals as examples of these concepts and discuss how evidence-based accessibility approaches can be used in a statistical consulting context. The focus of this paper is on communication and cognitive accessibility.

## Organizational framework for accessibility in statistical consulting centers

Discussions about effective statistical consulting and collaborations have traditionally focused on accuracy, efficiency, and methodological rigor. However, it is also important to recognize that clients and consultants have varied learning or communication preferences, and collaboration strategies should account for this throughout the consultation process. For consultants, the SCC environment involves navigating complex social dynamics with clients and colleagues, managing short- and long-term projects, and coping with overstimulating environments [2]. For clients, consultants support diverse learners by providing verbal and written explanations, illustrations, and preparing guidance documents and check lists to support their clients’ understanding and decision-making. This support is often provided on an ad-hoc basis to address emergent needs, rather than being systematically integrated into the process for all clients.

The UDL framework aims to support individuals of all abilities and learning styles, by providing multiple means of engagement, presentation, and action and expression [3–6]. Working under this framework, SCC consultants present information in different formats to accommodate different learning styles (“multiple means of representation”), to offer personalized engagement for different needs (“multiple means of engagement”), and to empower clients or themselves to apply what they know or learn in research projects (“multiple means of expression”).

SCCs can focus on ways to intentionally design accessible environments, processes and systems, since cognitive, emotional, and social capacities are subject to large variation. This is not limited to lifelong conditions but can also include temporary disabilities. For example, hearing loss due to an ear infection, eye strain from extended screen time, fatigue after illness, difficulty concentrating due to emotional challenges, or family obligations can affect everyone. A SCC could begin by developing a written accessibility policy for a multi-faceted, organizational framework addressing staff training, best practices for processes, accessible infrastructure (Table 1). Evaluation at regular intervals should be included in such a policy to identify areas for improvements. Staff training could be built into a SCC’s own customized program of professional development [7] if or by identifying accessibility resources provided by the university.

**Table 1.** Organizational framework for statistical consulting centers to support accessibility (SPIE)

Topics	Explanation
S – Staff training	Champion strategies for external and internal interactions by training consultants and supervisors in accessibility topics referencing guiding standards and providing administrative support. • Universal Design for Learning (UDL) guidelines [4] • digital accessibility Web Content Accessibility Guidelines (WCAG) [8] • effective communication guidelines [9] • accessible meetings [10] • professional ethics [11]
P – Processes and practices	Incorporate evidence-based practices in interactions (internal and external) • Incorporate accessibility guidelines in the pipeline from project initiation via intake forms, meetings, and presenting statistical information. • Employ UDL principles to address potential challenges for individuals, clients or consultants (Table 2). • Follow professional ethics standards
I – Infrastructure	Follow accessibility guidelines for tools and infrastructure • Conform to WCAG for the SCC website and adopt POUR principles [12] (perceivable, operable, understandable, and robust). • Accessible Meeting software (live captions, screen reader support) • Accessible document repositories (file formats, structure, alt text) must be accessible.
E – Evaluation	Improve practices and conform to accessibility guidelines • Periodic audits of the policy, infrastructure, and training • Document evidence of accessible processes and practices, including accessibility of deliverables for projects • Collecting feedback

**Table 2.** Effective communication and accessibility frameworks applied to statistical consulting environments

Executive function (individual level)	Potential challenges	Potential strategies based on UDL principles (system-level)
Attention	Staying focused during discussion; Difficulty processing rapid verbal exchange; auditory processing delays;	Multiple means of engagement: Provide detailed agendas Summarize key decisions in writing Written/asynchronous communication channels
Communication	Different levels of statistical literacy; diverse abilities; misinterpreting vague or indirect feedback; high emotional reactivity to perceived criticism; uncertainty about when to speak; organizing a narrative structure; High anxiety related to public speaking; sensory overload from the presentation environment (lights, sounds, audience).	Multiple means of representation: Presenting statistical concepts and analyses in multiple formats, for example, verbal explanation, report, checklist, graphics, video communication via different channels (meetings, email, collaborative documents, websites) Provide feedback that is direct, specific, behavioral, and written. Use a structured model like Situation-Behavior-Impact [13,14] Allow time for rehearsal, prepare back-up materials
Task Initiation	Delay in decision-making or next steps; difficulty with task initiation; feeling overwhelmed by large, abstract goals; challenges with time estimation.	Multiple means of action and expression: Provide checklist and timelines Break projects into small, concrete, sequential tasks using a visual Kanban board. Detailed checklists and Standard Operating Procedures templates for reporting; Processing the statistical content and presenting it in preferred formats
Management	Managing multiple datasets; managing deadlines and challenges with time estimation; Cognitive inflexibility (difficulty shifting analytical approach); hyperfocus on details leading to missed big-picture context;	Multiple means of action and expression: Use explicit start dates, due dates, and automated reminders. Provide Flexible scheduling, goal setting frameworks, Project management tools Have check-ins to review progress and discuss the “big picture” context and scope.

In a broader context, communication accessibility ensures that information is received and understood while cognitive accessibility supports comprehension and processing of information. Below we provide examples of evidence-based practices such as effective communication from initiating a project to presenting statistical information, as well as meeting modalities and web accessibility. These aspects are included in the SPIE framework, namely as part of processes and practices and of infrastructure with a call to increase awareness among staff. Below we provide accessibility resources and guidelines that could be leveraged in statistical consulting practices in such a framework. However, more work is needed to evaluate their effectiveness in a variety of settings and consulting scenarios.

## Effective communication

Consultants and clients work together to come to a shared understanding about the research aims, data structure, study design, analysis approaches, or planned tables and figures to be produced [15]. Shared understanding is critical for success in collaborative projects [16].

Detailed descriptions of statistical methods and concepts illustrated with step-by-step examples, presented in multiple formats (text, videos, illustrations) allow learners to choose what works best for them (Example 1). However, it may not be feasible for a SCCs to develop well-designed videos.

### Example 1: Accessible formats to explain statistical methods or concepts

Checklists simplify complex tasks by breaking them into manageable steps, supporting memory, and aiding task initiation and execution. Worked examples provide context and guide a user through steps. Visual aids can simplify complex ideas and reduce cognitive load. Author MH has been involved in developing materials to explain the importance and give guidance for initial data analysis (IDA). IDA is a crucial step for all data analysis projects to work with a data set and accurately address a research question. It is a systematic process to provide reliable knowledge about the data to determine its suitability and interpretability. Information about IDA is provided in multiple formats:

- Checklist: https://stratosida.github.io/assets/IDAchecklist_cross.pdf
- Example with R code: https://stratosida.github.io/regression-regrets/
- Paper: Regression without regrets https://doi.org/10.1186/s12874-024-02294-3
- Video: https://www.youtube.com/watch?v=VEJ8Xc7e6PI
- Zine: https://stratosida.github.io/assets/Zine_IDA.pdf

Checklists simplify complex tasks by breaking them into manageable steps, supporting memory, and aiding task initiation and execution. Worked examples provide context and guide a user through steps. Visual aids can simplify complex ideas and reduce cognitive load. Standardization of statistical reports can help ensure consultants provide high quality work and follow best practices and build trust and confidence. The use of well-designed intake forms [17] (Example 2), checklists for statistical analysis plans [18], and a statistical reporting template support consistent, high quality consulting experiences.

### Example 2: Intake form to initiate a statistical consultation

An intake form should provide sufficient information for the SCC to assign projects to consultants and should be simple enough so as not to overwhelm a potential client. A clear structure of an intake form with a few simple questions is helpful to improve accessibility. Besides including the typical questions such as contact, brief project description, deadlines, and acknowledgements of ethical issues, it should also include specific questions or optional choices to help with accessibility such as those related to preferred meeting format (e.g., in-person, online, or another), to communication style (e.g., written or verbal), or to personal forms of address (e.g., preferred name, pronouns).

Statistical software development has evolved to adoption of best practices in statistical graphics including color-blind friendly palettes [19] and adding contrast and redundant cues such as line styles, marker symbols, and labels. Recommendations for effective visual communication help with presenting information clearly [20]. Providing screen readable documents of statistical results can be achieved with many leading statistical and programming languages or platforms including R/RStudio, Python, Jupyter, VS Code, MATLAB, SAS, SPSS, Stata, and Julia [21]. The HTML output is easy to navigate and can show text explanations, figures, interactive figures, or code [22].

### Example 3: Accessible video content

Video content can be a valuable resource for communication. In contrast to live lectures or talks, videos may be watched at any time, rewatched, paused, rewound, and slowed down to aid understanding. Author MPW created a series of short videos introducing a statistical topic, measurement error, to a general audience. The videos are designed with various aspects of communication accessibility in mind. These include minimizing on-screen text to reduce reliance on English comprehension and providing space for captioning within the video design. Graphics employ a simple, clear design to aid visual accessibility and interpretation. Link: https://youtu.be/zFrvD6PUNg8?si=075xmp_wjouJntZt

However, some learners might prefer and benefit from added text in videos.

## Meeting modalities

To accommodate individual needs a statistical consulting center can offer different meeting modalities, such as one-on-one meetings, in-person or online. Benefits of online meetings include the faster turnaround for scheduling meetings and answering questions [23] and the ability to record the meetings for later review, since statistical consulting meetings can be densely packed with information. Enabling captions and transcripts allow for later review and better understanding of points that may have been missed during the consulting session. However, gauging understanding, explaining multiple approaches, or discussing strategies may be less effective in online meetings compared to in-person meetings and technical issues like lag disrupt the flow of conversation [23]. Zoom fatigue refers to feelings of exhaustion or anxiety associated with video conferencing [24]. In statistical consulting contexts, simple modifications, such as reducing the video window size or eliminating the self-view function, can alleviate the constant sense of self-surveillance, fostering a less stressful environment. Disabling their cameras accommodates individual attention spans and reduces the cognitive load associated with continuous visual processing. Addressing potential sensory overload by moderating screen brightness and simplifying backgrounds promotes sharper focus on substantive content.

## Web accessibility

Information about an SCC’s mission and resources can typically be found on a website. Web accessibility depends on several components working together. A SCC can play an important role in this process by checking compliance with accessibility standards, identifying barriers, and supporting the ongoing use of best practices to foster effective communication and collaboration among diverse audiences. SCCs with limited resources can improve web accessibility using free tools like Web Accessibility Evaluation Tool [25], simple checklists, accessible website themes, and user feedback. The Web Content Accessibility Guidelines (WCAG) 2.2 consist of guidelines with four main accessibility principles, known by the acronym “POUR:” perceivable, operable, understandable, and robust [12]. In statistical consulting, visualizations like charts or images should be accompanied by text (Perceivable principle). For example, a bar chart summarizing survey results should include a text description of main findings or trends for screen reader users. Accessible web reports, dashboards, or data portals should include clear page titles, logical heading structures, skip navigation links, and consistent labels along with descriptive link text to ensure users with disabilities can navigate and understand the content effectively (Operable principle). While some clients may find search features more accessible than using a hierarchical navigation menu, this preference can vary. Offering multiple navigation options can benefit all visitors to the SCC’s website. Content and interfaces should be clear, predictable, and easy to follow for all users (Understandable principle). This is particularly important when communicating complex data, analyses, or recommendations to a diverse audience. A website’s written content should be accessible by people with cognitive, sensory, and communication differences, including those who use assistive or alternative communication tools. This involves explaining statistical terms clearly, avoiding the use of confusing or unnecessary language in reports. Assistive technology tools like text-to-speech or translation software may interpret abbreviations inconsistently unless spelled out (Readable principle). In statistical consulting, this means that data dashboards, interactive reports, and online tools should be built so that screen readers and other assistive devices can accurately convey the content. Assistive devices interpret graphical and interactive web content by reading the underlying code and semantic structure rather than the visual layout. All charts and images should include alternative or descriptive text, for example: “Bar chart shows sales increasing from $10K in January to $25K in June.” (Robust principle). Adhering to these accessibility standards supports clearer communication and engagement for potential users with or without situational limitations (e.g., slow internet connections or environments where audio cannot be used) or other limitations (visual, audio, physical, cognitive).

## Accessibility for neurodivergent individuals in statistical consulting

We discuss accommodations for communication and cognitive accessibility for neurodiverse individuals as this covers a broad spectrum. According to the National Library of Medicine *“Neurodiversity describes the idea that people experience and interact with the world around them in many ways, with no one ‘right’ way of thinking, learning, and behaving, and differences are not deficits”* [26]. Neurodivergent individuals may exhibit one or more neurological variations, such as attention deficit hyperactivity disorder, autism spectrum disorder, dyslexia, gifted, and others [27]. They may exhibit deficits in communication and social behaviors compared to neurotypical individuals [2,28]. These are not static experiences, not always visible, or immediately present. However, the strength-based approach to neurodiversity reframes neurodiverse characteristics as valuable variations, such as attention to detail, pattern recognition, visual thinking, or creativity [29]. Accommodations for cognitive and communication accessibility can leverage the strengths of neurodiverse individuals and help them thrive in their environments [28,29]. All hypothetical situations described below are based on our experiences working with consultants and clients.

Potential challenges for consultants and clients in a statistical consulting environment are listed in Table 2 and mapped to executive function at the individual level and UDL framework at a system level. The strategies are designed to remove environmental and procedural barriers so that diverse learners are supported and can operate at the full capacity of their analytical strengths.

## The neurodivergent client

In this section we focus on specific challenges of communication and cognitive accessibility for neurodivergent clients in SCCs.

Executive function can be challenging for neurodivergent individuals, including motivation, emotional regulation, organization, sustained effort, focus, and memory. A neurodivergent client may be consciously or subconsciously suppressing their natural behaviors (“masking”). However, suppression can lead to burnout, intense physical, emotional, and psychological fatigue, or lower self-esteem [30]. This may not be obvious to the consultant. In contrast, if the behaviors are not suppressed, it can be distracting to a neurotypical consultant who is unaware of these challenges for their clients.

Effectively supporting a neurodivergent client begins with establishing clear structure and flexible communication channels. Setting explicit expectations and providing an agenda at the start of each meeting creates a predictable and manageable framework. This structural support should be complemented by offering multiple means of communication to aid information processing. Because some clients prefer verbal explanations while others absorb written information more easily, providing both formats is often essential for comprehension [31]. Clear, unambiguous language that does not require the need to make inferences and conciseness without unnecessary detail reduces cognitive load [31]. This clarity can be enhanced by providing step-by-step instructions that separate large tasks into smaller, more manageable components. Consultants should be aware that open-ended questions can be frustrating, making more direct or closed-ended questions a preferable alternative.

Individuals process sensory information, such as noise, light, odors, or movement, differently [31]. A client may find eye contact overwhelming or distressing, leading to avoidance. Providing fidget toys is increasingly common at meetings and may help the client’s comfort level and enhance engagement and attention. Offering flexibility in meeting times to align with an individual’s preferences is a useful accommodation. Just as it is with neurotypical clients, it is important to empower neurodivergent clients, making them feel valued, and supporting their scientific growth [5].

### Situation 1: Workstyles of neurodivergent clients

Some graduate students may prefer a structured process with detailed task lists at a consultation. Consultants can schedule regular meetings where students may implement analyses alongside the consultant and develop skills and confidence during these sessions [32]. Such sessions may be followed with periods of independent work. Consultants can also help the students to develop realistic timelines if students feel discouraged by not being able to sustain long hours of concentrated work or being unable to work as much as they have been advised to do.

### Situation 2: Communication strategies for anxious clients

Graduate students may present with anxiety at a consultation when asked open-ended questions and providing them comments to prompt them to think critically about their project. An anxious student may find this frustrating [33]. When this is evident, a shift in approach to providing calm interactions, letting them set the pace, nudging them about progress during periods of idleness may help the student. Providing step-by-step guidance on statistical methods or data management can result in more positive and productive responses.

This highlights the need to recognize the barriers due to dealing with emotions and addressing this by shifting approaches to help reduce cognitive load and being flexible with pace [6]. Consultants should examine their assumptions about the background of clients and be flexible to adapt approaches to the clients’ needs quickly. However, even with flexible policies, there are limitations for a busy SCC due to available time or resources.

Strategies and sample language, grouped by meetings, deliverables, or building researchers’ capacity, are provided in Table 3. They could be used by consultants for their interactions with clients to create a welcoming environment and set clients at ease regardless of their learning and communication styles.

**Table 3.** A consultant’s strategies for helping clients

Executive function	Sample questions for consultants	Potential strategies
Meetings		
Communication	“What is your preferred meeting modality?” “How do you prefer to communicate (email, video calls, written reports, meetings)?” “Feel free to turn your camera off or use the chat to ask questions.”	Offer online meetings and assure audio and screen sharing work well. It is OK for the client to have the camera off or to communicate via the chat feature.
Communication	“Would you like to record our meeting?” “How would you like to ask questions or provide comments? (e.g., discussion, written comments, collaborative documents)”	Allow recording and captions.
Management	“What is your preferred meeting modality, online or in-person? We also have walk-in office hours….”	Offer office hours where clients can work independently and ask questions when needed.
Attention	“Please let me know if I am explaining too fast.”	Offer breaks. Offer to repeat information.
Attention	“Please let me know if you have any questions.” “Are there publications with related results?”	Encourage feedback and questions. Asking clients to make connections to their research domain.
Attention		Provide step-by-step guidance to explain the statistical methods and why this is needed to address the research objectives
Task initiation		Send a follow-up email with a summary of the meeting.
Deliverables		
Task initiation	“Would you like to discuss a checklist of next steps?” “Here are two action items to do before our next meeting. Does this work for you?”	Provide goals or next steps in small, manageable tasks. Explain reasons.
Communication	“Would it help if I explained this another way?”	Provide alternative forms of explanations, verbally, visually or written. Demonstrate use of statistical software.
Communication	“Would you prefer a graphic, table, or text in the report?” “How do you prefer information to be presented? (e.g., written summaries, verbal report, tables, data visualization, interactive graphics)”	Provide statistical results in a written report or go over it verbally. In figures, use color, layout, and annotations to emphasize trends, outliers, or actionable insights
Attention	“These references provide further detail about…”	Provide resources to understand methods and their applications and build clients’ knowledge and capacity.
Empowering the researcher		
Attention	“To illustrate why this approach is appropriate, please consider….”	Provide examples from client’s research domain. Tailor statistical approaches to align with what matters most to the client.
Task initiation	“Would you like to discuss how you can use this template for your project?”	Provide tools or templates for clients’ own decision-making. Help with developing strategic approaches.
Communication	“These references provide further detail about…”	Provide resources in multiple forms (e.g., videos, papers, books, software instructions)
Management	“Would you like me to outline the next steps and possible timelines?”	Suggest timelines for manageable tasks.

## The neurodivergent consultant

The academic research environment, particularly in a quantitative field like statistical consulting, demands deep focus, pattern recognition, and logical thinking – which are common neurodivergent strengths. It also involves navigating complex social dynamics with clients and colleagues, managing short- and long-term projects, and coping with overstimulating environments [2]. In this section, we focus on how to help neurodivergent consultants and their supervisors navigate these challenges.

Executive functions are the higher-order cognitive processes required for planning and executing goal-directed behavior. The performance of a highly intelligent neurodivergent consultant is often mediated not by their analytical ability, but by the executive functions required to deploy those abilities effectively over the course of a complex project [34]. A common challenge is the difficulty in breaking down large, abstract goals (e.g., a grant proposal) that may lead to a feeling of being overwhelmed and result in procrastination into a manageable sequence of concrete, actionable tasks. Challenges in working memory, namely the ability to hold and mentally manipulate multiple pieces of information at once, can affect the individual’s ability to shift between tasks, adapt to unexpected changes in a research plan, or consider alternative analytical strategies when an initial approach is unsuccessful. Besides navigating task-oriented behaviors, consultants also engage in relationship-oriented tasks [2]. A neurodivergent consultant working with a neurotypical client may experience negative perceptions. Social communication with neurodivergent individuals differs from neurotypical norms in processing and expression, and this is sometimes thought of as a lack of desire for social connection [35]. These include a tendency to interpret language literally, making it difficult to understand sarcasm, idioms, metaphors, and other forms of indirect speech. Challenges in reading and interpreting non-verbal cues such as body language, facial expressions, and tone of voice, and slower auditory processing, can result in missing details or lead to misunderstandings in collaborative meetings.

Addressing these needs is a prerequisite for enabling a consultant to access their higher-level skills (Table 2) [36–38]. A consultant can advocate for themselves by talking to their clients about communication and work preferences to help set expectations and build trust. For example, “I may need time to respond to questions as I like to reflect before answering,” “I may need to turn off the camera in the Zoom meeting so I can focus better,” or “I prefer short online meetings and follow-up with comments or additional questions” helps set expectations. Giving permission to clients or supervisors to be honest and upfront can avoid misunderstandings and sets the stage for successful, long-term collaborative relationships. For example, “I appreciate clear and direct feedback to make sure I can deliver best results” or “Please feel free to get in touch if you have not heard back by […].”

Supervisors may have unrealistic expectations or assumptions regarding workstyles, timelines, schedules, productivity, or communication of neurodivergent consultants [27,28]. However, not being able to deliver timely results holds up projects and hurts the reputation of the SCC, since dependability establishes trust and cultivates long-lasting relationships. Providing executive function support in management and task initiation can help consultants to meet the needs of the SCC. Supervisors can successfully engage with consultants, with regular, predictable check-ins, providing supportive feedback, clearly communicating expectations, and allowing flexible schedules. They are advocates and create networking opportunities to help with career growth. Fostering an understanding of the SCC’s strategic goals, inclusive leadership and seeking input for organizational policies, and discussing a consultant’s career path tailored to their strengths helps the consultant’s professional development and achieve performance goals. Providing a respectful environment where differences are normalized and welcomed creates a sense of belonging for everyone.

### Situation 3: Adaptive work strategies for consultants in a supportive environment

A statistician often worked hard to mask any differences to their neurotypical peers. Connecting to other neurodivergent individuals helped them recognize similarities in their experiences and reframe their own challenges – not as personal failings, but as mismatches with neurotypical work expectations. At the SCC, they struggled with juggling multiple concurrent projects. Time and project management were difficult, and feedback from supervisors triggered anxiety. By asking advice from supervisors and colleagues, drawing on experiences of neurodivergent peers, institutional support groups, or resources on neurodivergence such as podcasts, they experimented with strategies until they found approaches that suited them. By blocking time on their calendar, they overcame the barrier of responding to client emails, turning a challenge of executive dysfunction into a structured routine. When hyperfocus risked them being consumed by overwhelmingly many options, scheduled check-ins with mentors helped them recalibrate scope and redirect effort. Flexible work hours enabled them to align their tasks with natural productivity rhythms. Clear and constructive communication with supervisors eased their anxiety when receiving feedback. Framing suggestions as strategies for improvement rather than focusing on deficits helped them develop confidence in professional settings. With their explanations and comprehensive solutions to complex research questions they are appreciated by clients. With their thoughtful contributions in staff meetings, they are highly valued members of the team.

This highlights the importance of the SCC creating a culture that values differences as assets, whether in background or work styles. Flexible scheduling, mentorship that emphasizes growth and experimentation, and constructive feedback framed around strategies for improvement help neurodivergent consultants thrive.

## Discussion

SCC can facilitate accessibility by building an inclusive environment through adopting policies that include training and mentoring to increase awareness among their staff. Tools and infrastructure should conform to accessibility guidelines. Accessibility practices and processes and tools should be evaluated periodically for continuous improvement. We introduce an organizational framework (SPIE) and hope to stimulate discussion, inform practice, and develop further evaluation and refinement among the statistical consulting community. Future studies are needed to evaluate strategies in different scenarios.

Many researchers do not disclose their personal circumstances [39]. A welcoming environment empowers consultants and clients to ask for accommodation that helps them achieve success to meet the statistical demands of their research projects. Consultants can support their clients through multiple strategies that enhance clarity and understanding of statistical approaches. This can help them avoid feeling overwhelmed or isolated [27]. Unsolicited feedback from clients at our SCC, some anonymous, highlight several themes that fit into the framework of UDL of representation, engagement, and expression. Explaining clearly and building clients’ statistical knowledge through teaching methods and sharing resources, beyond providing results of analyses, was much appreciated. Many clients noted that they felt more confident and capable after working with their consultant. Clients praised their consultants for their quick response, reliable follow-up, and availability outside of scheduled meetings when needed. A friendly, respectful, and professional demeanor fostered trust and results in enjoyable, successful collaborations. Everyone benefits when accessibility principles are applied uniformly, whether neurodivergent or neurotypical individuals. For example, offering flexible meeting times helps a neurodivergent client with delayed sleep phases or a neurotypical client with childcare responsibilities. Adopting these strategies may lead to collaborating with a wider range of individuals, empowering clients and consultants, and resulting in satisfactory completion of research projects, publications, dissertations, or successful grant proposals.

There are barriers to adopting these strategies. Steps for improving accessibility take time, strategic planning, and funding. The SCC may have insufficient institutional support to make sure their digital resources, project management tools, and infrastructure conforms to accessibility guidelines. Consulting projects for clients needing extra time may potentially exceed the allotted time limits per SCC policy or partnership agreements [40]. Developing standardized reporting tools, visual aids, supportive materials, and templates takes time. However, these lead to consistency in providing high-quality consulting and save time once established. The goal should always be to ensure a client is provided with a consultancy experience that optimizes accessible accommodation. A suboptimal attempt at accommodation may be worse than no attempt. An example of this is automated captioning, which is often viewed as “better than nothing” but it may not be of a good enough quality, and no attempt is made to correct them. A mindset of doing something poorly serves as a barrier or disincentive to doing something well. This work provides a structured starting point and call to action for the statistical consulting community to explore organizational practices that support accessibility and their impacts. Physical, institutional, and financial accessibility is outside the scope of this paper and needs further work.

A statistical consulting center can champion strategies by training consultants and supervisors in accessibility topics and providing administrative support. The environment can stimulate neurotypical or neurodivergent consultants and enable them to excel. Investing in professional development, new statistical methodology, new technology, or scholarly products designed to communicate the findings to diverse audiences is crucial [40]. Building a welcoming community, taking time for empathy and understanding to assess specific needs, creates a sense of belonging, improves experiences and builds trust and confidence in clients and consultants alike, and leads to successful outcomes.
